# Ultrasound combined with hysteroscopy for optimum treatment of Robert’s uterus: a case report and a review

**DOI:** 10.1186/s12905-022-01903-x

**Published:** 2022-08-07

**Authors:** Ying Liu, Chenxiao Hou, Yingjie Zhou

**Affiliations:** Department of Obstetrics and Gynecology, The Second Hospital of Hebei Medical University, 215 Heping West Road, Shijiazhuang, 050000 Hebei China

**Keywords:** Hysteroscopy, Laparoscopy, Malformation, Robert’s uterus, Ultrasound

## Abstract

**Background:**

Rbert’s uterus, also known as asymmetric septate uterus, is a rare genital malformation first reported by Dr. Robert in 1970. Robert’s uterus is characterized by a septate uterus with a blind hemicavity and an intact external fundus. According to some reports, Robert’s uterus was typically managed by laparoscopic uterine resection of a hemicavity, laparoscopic endometrectomy, and even hysterectomy. Considering that fertility preservation is important in young patients, we recommend ultrasound-guided hysteroscopic septum resection as an optimum treatment for Robert’s uterus.

**Case presentation:**

Herein is described a clinical case of Robert’s uterus in a 15-year-old girl who was misdiagnosed for primary dysmenorrhea in the beginning. Magnetic resonance imaging (MRI) and 3-dimensional (3D) ultrasound identified an asymmetrical uterine septum. The patient was treated using ultrasound-guided hysteroscopic treatment without laparoscopy. The surgical procedure lasted less than an hour, and the symptoms of dysmenorrhea were relieved during a six months follow-up.

**Conclusions:**

Ultrasound-guided hysteroscopic septum resection is the preferred treatment for Robert’s uterus.

## Background

Robert’s uterus is a rare type of müllerian duct anomaly due to obstruction by the asymmetric septum. Only a few cases have been reported in the recent years, and it has not been adequately classified with the American Fertility Society (AFS) system [1]. The uterine cavity connected to the cervix can only see one side of the fallopian tube ostium, at the same time, the other side of the uterine cavity is blind. Patients often complain of recurrent abdominal pain and severe dysmenorrhea ipsilateral to the lesion after menarche. Currently, the diagnosis of Robert's uterus is mainly identified by MRI and 3-dimensional (3D) ultrasound (US). This malformation may be misdiagnosed as a two-horn uterus or a one-horn uterus with one side of the stump. The key points of differential diagnosis are the separation distance of the uterus fundus and whether the fundus is close to normal. Following an updated embryological and clinical classification of female genital tract malformations, Rober’s uterus was caused by müllerian duct anomaly but without renal agenesis which was also reported in the literatures [2]. Misdiagnosis can result in persistent symptoms and inappropriate treatment. We emphasize the role of ultrasound and hysteroscopy in this rare intrauterine procedures. Real-time surgical ultrasound can simultaneously display the uterus and surgical instruments, thus it is possible to improve the effectiveness and safety of surgical intervention. Ultrasound should be used more in the operating room to guide various operations to reduce intraoperative risks and complications. Frequent use of ultrasound improves training opportunities for trainers and clinicians.

## Case presentation

**Fig. 1:**
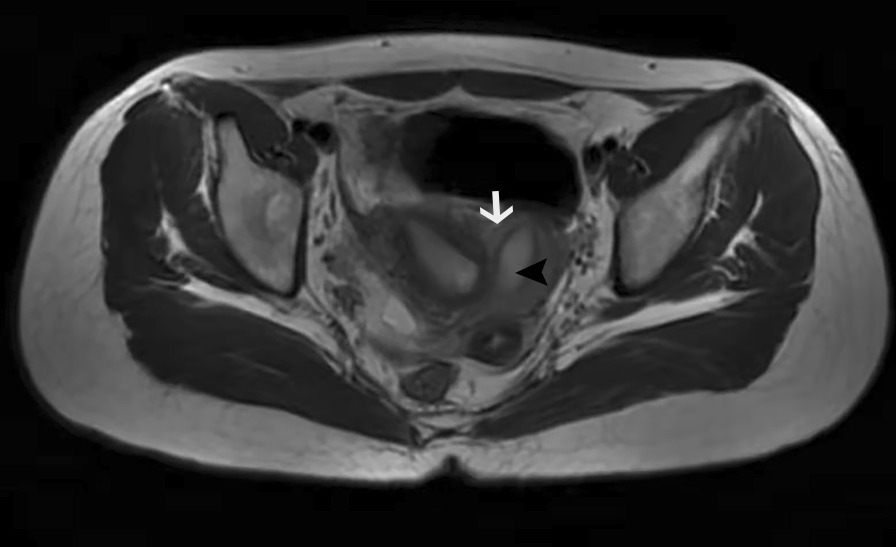
MRI image showing the left uterine cavity (black arrow) communicating with the cervix and irregular fundus(white arrow)

**Fig. 2.**
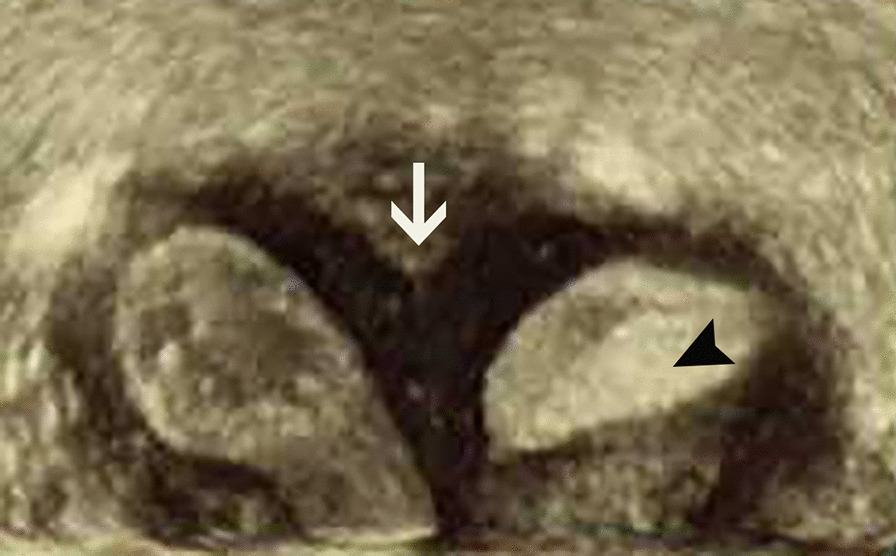
3D ultrasound showing asymmetric uterine septum with a closed right uterine cavity (black arrow) and slightly sunken fundus (white arrow)

Here, we report the case of a female patient initially misdiagnosed for primary dysmenorrhea. A 15-years-old woman suffering from dysmenorrhea with regular menstruation was referred to our department. She complained of dysmenorrhea in the past year, mainly in the right lower abdomen, accompanied by nausea, vomiting, and fever. Pain medication had to be regularly taken during menstruation. After menstruation, the pain was relieved, and the temperature returned to normal. There was no special treatment because the preliminary local pelvic 2-dimensional (2D) ultrasound showed no abnormalities in the uterus. After coming to our hospital, no apparent abnormalities were found in the gynecological physical examination. MRI performed in our department showed a single-horned uterus (Fig. 1). The residual horned uterus did not appear to be connected to the uterine cavity. Our central 3D ultrasound showed that the bottom of the uterus was slightly depressed, and the right uterine cavity was not connected to the cervix. Still, the endometrial was visible, and the left uterine cavity was connected to the cervix (Fig. 2). The patient had no symptoms of urinary abnormalities and ultrasound of the urinary system showed that both kidneys were normal. Hence, the patient was diagnosed with Robert's uterus and scheduled for hysteroscopic treatment combined with ultrasound.

**Fig. 3:**
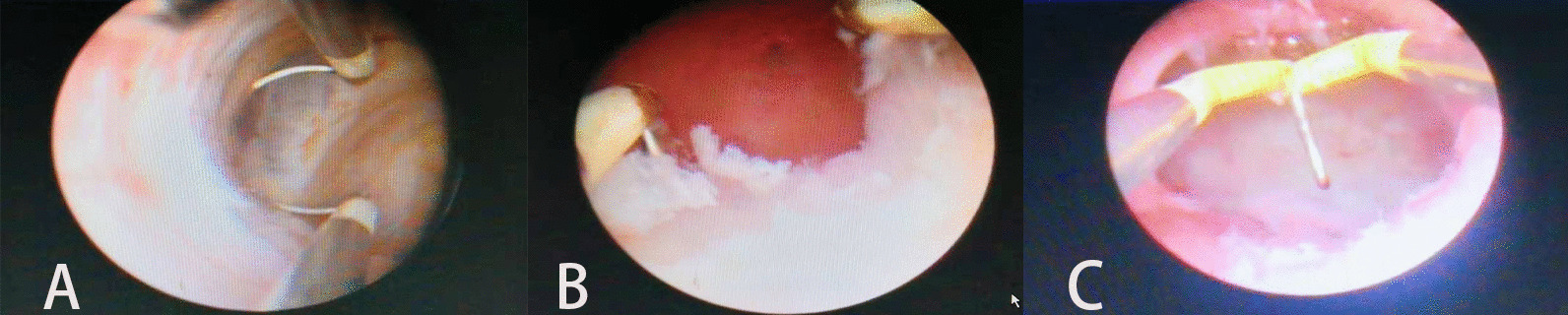
Image of hysteroscopic surgery **A** Asymmetric uterine septum visible under hysteroscopy. **B** Right fallopian tube opening revealed after mediastectomy. **C** Complete uterine cavity after hysteroscopy

**Fig. 4:**
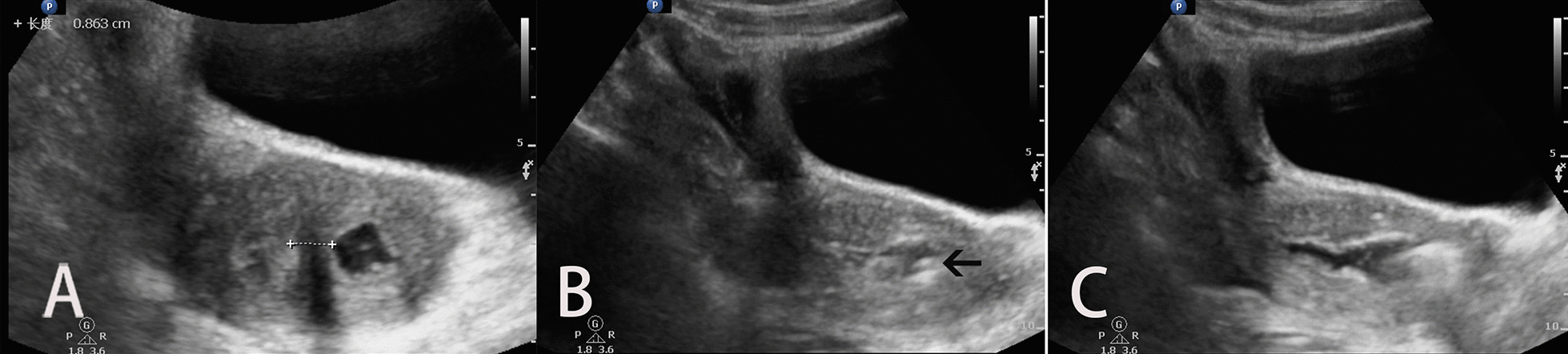
Ultrasonic monitoring picture **A** Only the filling of the left uterine cavity could be seen on the ultrasound, and the asymmetric uterine septum could be clearly shown and measured in this section. **B** Real-time display of electric hook position under bladder filling (black arrow). **C** Abdominal ultrasound monitoring of uterine cavity communication

During the hysteroscopic operation a normal-sized vaginal and cervix were seen under the hysteroscopic lens. Abdominal ultrasound can guide the doctor dilated into the uterine cavity with the expansion rod in real time. Under ultrasonic display, the uterus was clearly exposed by the filling bladder. After hysteroscopic electroacupuncture entered the uterine cavity, the position of the electroacupuncture could be observed on the ultrasonic display. The lowest point where the septum was incised starting from was identified by abdominal ultrasound. The starting point was confirmed, the hysteroscopic electric needle was cut into the right uterine cavity from the left side, and blood outflow was visible. During the operation, the uterine cavity and myometrium were dotted with strong echoes due to thermal effects, but the integrity of the uterine contour could still be seen on the monitor. After the uterine septum was displayed on both sides, the uterine septum was removed from the lowest point of the uterine septum to the bottom of the uterus. A successful unification of the two sides was finally recorded (Fig. 3). Both oviduct openings were seen under hysteroscopy (Fig. 4). During a six months follow-up, dysmenorrhea disappeared.

## Discussion and conclusions

Robert’s uterus, or asymmetric uterus, is an uncommon uterine müllerian anomaly first reported by Dr. Robert in 1970[3]. Ultrasound plays a key role in reproductive medicine diagnosis. Deenadayal et al. reported that a well-trained and dedicated ultra-sonographer could measure the uterine septum thickness, the outer contour of the uterus, and pelvic endometriosis using modern 3D US technology [4]. In their opinion, 3D US is better in providing a correct diagnosis and intraoperative monitoring. Most reports of Robert's uterus are case reports for which treatment has not been established. Initially, laparotomy was the treatment choice for Robert's uterus since the 70 s. Rebelo et al. reported the case of a 17-year-old patient who underwent a right hemi-hysterectomy to protect fertility instead of the previously recommended total hysterectomy [5]. Capito et al. simultaneously performed a complete endometrectomy and myometrium reconstruction on a 15-year-old woman diagnosed with Robert's uterus [6]. Vural and colleagues diagnosed a 24-year-old patient with Robert's uterus who underwent a laparotomy. After endometrectomy was performed through a hysterotomy incision, she got pregnant. However, a cesarean section was performed during birth due to combined placenta implantation [7]. The early procedure would have caused serious damage to uterus, postoperative scar pregnancy and placenta implantation. To avoid overtreatment, Robert's uterus should be distinguished from a bicornuate-unicollis uterus, which might be requires a hemi-hysterectomy.

Hysteroscopic metroplasty has become an accepted procedure for the treatment of the uterine septum as the endometrial damage is small, scar formation is avoided, and the operating time is short. In recent years, hysteroscopic treatment of Robert's uterus has generally been performed under laparoscopic monitoring [8]. We believe that ultrasound plays an irreplaceable role in the diagnosis and treatment of Robert's uterus. Some studies have performed a comparison of the ultrasound-guided and laparoscopic-guided hysteroscopies for metroplasty. Vigoureux et al. showed that hysteroscopic with abdominal ultrasound guidance has advantages in both short-term and long-term postoperative complications, such as perforation and persistent septum [9].

In our abdominal ultrasound-guided hysteroplasty case, the bladder was filled to expose the fundus of the uterus. Our experienced sonographer could see the dilation path in real time during cervical dilation. Hysteroplasty requires the surgeon to move up from the lowest end of the asymmetric diaphragm. Hysteroplasty maximizes the uterine cavity, and the remaining fundus muscle layer should be greater than 1 cm. All of these requirements can be achieved with abdominal ultrasound guidance. It should be noted that the absence of laparoscopy occurred only when there was no endometriosis in the preoperative evaluation. Considering that preoperative MRI and ultrasound did not indicate ovarian endometriosis in our case, we designed a therapeutical strategy of ultrasound-guided hysteroscopic surgery with the patient's parents permission. We believe that hysteroscopic hysteroplasty guided by abdominal ultrasound is necessary even in the presence of preoperative pelvic endometriosis. Abdominal ultrasound guidance can compensate for the deficiency of laparoscopic guidance and obtain real-time mediastinal resection information. Women with known intrauterine pathologic factors should undergo ultrasound-controlled surgical hysteroscopy to avoid unnecessary laparoscopy.

## Data Availability

All data generated or analysed during this study are included in this published article.

## Abbreviations

MRI: Magnetic resonance imaging
3D: 3-Dimensional
2D: 2-Dimensional
US: Ultrasound
AFS: American Fertility Society
